# Immunological Manifestations in *GALE* Deficiency: Extending the Spectrum Beyond Thrombocytopenia and Galactosemia

**DOI:** 10.1007/s10875-026-02018-w

**Published:** 2026-04-16

**Authors:** Eyal Kristal, Aya Khalaila, Mahdi Asleh, Abed Abu Quider, Yotam David Eshel, Naim El Mahdi, Nurit Hadad, Shlomo Almashanu, Hagit Miskin, Orna Staretz-Chacham, Miriam Ben-Harosh

**Affiliations:** 1 Pediatric Ambulatory Center, Saban Children’s Hospital, Soroka University Medical Center, Beer Sheva, Israel; 2 Pediatric Hematology and Oncology Department, Saban Children’s Hospital, Soroka University Medical Center, Beer Sheva, Israel; 3 Pediatric Allergy and Immunology Clinic, Saban Children’s Hospital, Soroka University Medical Center, Beer Sheva, Israel; 4 Pediatric Metabolic Clinic, Saban Children’s Hospital, Soroka University Medical Center, Beer Sheva, Israel; 5 Clalit health services, Tel Aviv-Yafo, Israel; 6 Flow Cytometry Unit, Soroka University Medical Center, Beer Sheva, Israel; 7 Israeli National Newborn Screening Program, Ministry of Health, Jerusalem, Israel; 8 Joyce & Irving Goldman Medical School, Faculty of Health Sciences, Ben-Gurion University of the Negev, Beer Sheva, Israel

**Keywords:** *GALE*, Congenital disorder of glycosylation, B-cell, Immunodeficiency, Thrombocytopenia

## Abstract

**Background:**

Mutations in the *UDP-galactose 4’-epimerase* (GALE) gene disrupt the Leloir pathway of galactose metabolism and play a key role in protein and lipid glycosylation by providing essential nucleotide sugar donors. While GALE deficiency is known to cause galactosemia and thrombocytopenia, its impact on the human immune system remains largely unexplored. We aimed to characterize a novel immunodeficiency phenotype associated with the homozygous p.R51W GALE variant.

**Methods:**

We performed a comprehensive immunological evaluation of seven pediatric patients (aged 2.5–10 years) homozygous for the p.R51W GALE variant. Assessments included immunoglobulin levels, vaccine-specific antibody responses, lymphocyte subsets, and kappa-deleting recombination excision circles (KRECs).

**Results:**

All patients exhibited profound B-cell lymphopenia (0.06–0.12 × 10^3/µl). While KREC levels were detectable, absolute counts (6–50 copies/µL) were significantly below the age-specific 5th percentile, suggesting a quantitative defect in central B-cell output. Detailed B-cell phenotyping demonstrated preservation of the full spectrum of maturation stages, indicating no absolute block in differentiation. Serum IgA and IgM were consistently low, whereas IgG levels were preserved or elevated, driven by a paradoxical increase in the IgG3 subclass. Specific antibody responses were impaired. Clinically, patients suffered from recurrent viral respiratory infections but lacked invasive bacterial disease.

**Conclusion:**

The p.R51W GALE variant results in a distinct hematopoietic stress phenotype characterized by significant quantitative B-cell deficiency. The absence of a clear differentiation block suggests that lymphopenia results from reduced central B-cell output combined with impaired peripheral homeostasis. We propose that the underlying GALE-mediated glycosylation defect compromises B-cell homeostasis. These findings identify GALE deficiency as a novel cause of primary immunodeficiency and suggest that immunological screening is warranted in affected patients.

**Supplementary Information:**

The online version contains supplementary material available at 10.1007/s10875-026-02018-w.

## Introduction

UDP-galactose 4′-epimerase (*GALE*) is an essential enzyme in galactose metabolism. GALE catalyzes the final, reversible step of the Leloir pathway of galactose metabolism, interconverting UDP-galactose and UDP-glucose [1]. This interconversion is fundamental not only for energy production but also for the synthesis of glycoproteins, glycolipids, and proteoglycans, which are critical components of cellular structures and signalling pathways [2]. *GALE* activity is especially critical during early development and in rapidly proliferating tissues, including the hematopoietic and immune systems [3, 4].

Pathogenic variants in the *GALE* gene have historically been associated with type III galactosemia (OMIM #230350), characterized by variable clinical manifestations ranging from benign to severe systemic disease [1, 3, 4]. However, the genotype-phenotype correlation in *GALE*-related disorders is complex and incompletely understood. Not all patients with *GALE* mutations exhibit biochemical evidence of galactosemia or develop the classical clinical features of galactose intolerance such as cataracts, developmental delay, or liver dysfunction [5–8]. This phenotypic heterogeneity suggests that *GALE*’s functions extend beyond galactose metabolism, potentially influencing multiple cellular processes through its role in glycosylation pathways. Notably, patients in our cohort exhibited abnormal newborn screening (elevated galactose-1-phosphate on dried blood spots) but lacked clinical features of Galactosemia.

In recent years, a specific *GALE* variant has been identified in patients with chronic thrombocytopenia, often without evidence of galactosemia [9]. These variants appear to affect megakaryocyte development and platelet production through mechanisms distinct from galactose toxicity. The resulting thrombocytopenia in these patients is typically moderate to severe, with platelet counts ranging from 20 to 100 × 10^9/L. The bleeding tendency is variable and may not correlate directly with platelet count, suggesting qualitative platelet defects in addition to quantitative reduction [10].

The association between *GALE* deficiency and thrombocytopenia highlights the enzyme’s critical role in protein glycosylation, a post-translational modification essential for proper protein folding, stability, and function [10, 11]. Glycosylation affects numerous proteins involved in hematopoiesis, including cell surface receptors, adhesion molecules, and cellular signalling [12]. Consequently, *GALE* mutations likely represent a subtype of congenital disorders of glycosylation (CDG), a heterogeneous group of genetic diseases characterized by defective synthesis, attachment, or processing of glycans [11].

The importance of proper glycosylation for immune cell development, antibody structure, and function is well established [13]. Immunoglobulins are heavily glycosylated proteins, and these glycan structures significantly influence antibody effector functions, including complement activation, Fc receptor binding, and in vivo half-life [14, 15]. Data on immunological manifestations in patients with *GALE* mutation are sparse. Markovitz et al. [16] previously described one patient with a *GALE* mutation who exhibited lymphopenia, predominantly characterized by a low B-cell number and altered immunoglobulin production.

In this study, we characterize the immunological, hematological and clinical phenotype of seven pediatric patients with thrombocytopenia due to a confirmed *GALE* pathogenic variant.

## Methods

### Patient Selection

This study included molecularly confirmed patients, all homozygous for the same pathogenic variant in the *GALE* gene c.151 C > T (NM_001127621). These patients were followed at Saban Children’s Hospital, Soroka University Medical Center. Data were obtained from electronic medical records and included demographic, clinical, and laboratory data. The immunological evaluation was performed during routine outpatient visits when patients were free of acute infection. All patients except one (parental refusal) received vaccinations as per the mandatory schedule, including diphtheria-tetanus-pertussis, conjugated pneumococcal, and measles, mumps, rubella, and varicella (MMRV) vaccines.

### Immunological Workup

The workup included serum concentrations of IgG, IgM, IgA, and IgE; specific antibodies (anti-tetanus, anti-pneumococcal, anti-measles); lymphocyte subsets by flow cytometry; and Kappa-deleting recombination excision circles (KREC)(see Supplementary Information for details).

### Ethical Approval

Patients’ legal guardians provided written informed consent for inclusion in this study, which was approved by the institutional ethics committee (0250-04-SOR).

## Results

### Patient Characteristics and Clinical Findings

We identified seven pediatric patients (designated P1–P7) homozygous for the pathogenic variant in the *GALE* gene. All patients were identified through the Israeli National Newborn Screening (NBS) program due to elevated galactose-1-phosphate (Gal-1P) levels (Supplementary Information). Given that GALE is also essential for proper glycosylation, we screened for a glycosylation disorder via serum transferrin isoelectric focusing (IEF), which was normal in all patients (Table S1). All patients were initially diagnosed with *GALE*-associated thrombocytopenia, but further immunological and hematological evaluation revealed additional abnormalities (Table 1). The most common infectious manifestations included recurrent hospitalizations due to respiratory viral infections, predominantly respiratory syncytial virus (RSV) (4/7 patients) and influenza virus (3/7 patients). Patient P6 additionally presented with a methicillin-resistant *Staphylococcus aureus* (MRSA) skin abscess. Notably, none of the patients received prophylactic antibiotics or IVIG (immunoglobulin replacement therapy) during the study period. Hospitalizations for infections were typically brief (3–5 days) and responded well to standard acute care, with no recorded episodes of invasive bacterial disease or sepsis.

**Table 1:** Hematological and clinical characteristics

Age (years)	P1	P2	P3	P4	P5	P6	P7
	5	2.5	5	3	10	4	6
Infections	Flu A	Parainfluenza	RSV	RSV	RSV	RSV	Flu A
	hMPV	Flu A			hMPV	Parainfluenza	RSV
		Flu B				Covid	
						MRSA skin abscess	
Hb (gr/dL) MCV (Fl) LYM (⋅10^3^cells/µl) NEUT (⋅10^3^cells/µl) PLT (⋅10^3^cells/µl) MPV (Fl)	7.8 > 90 0.84 0.43 4 > 15	8.1 > 90 0.8 0.35 18 > 15	9.1 > 90 0.78 0.53 6 > 15	8.1 > 90 0.78 0.45 15 > 15	7.5 > 90 0.80 0.54 5 > 15	7.6 > 90 0.850 0.600 10 > 15	7.5 > 90 0.70 1.2 12 > 15

P, patient

Hb, LYM, NEUT, and PLT values represent each patient’s lowest recorded measurement

MCV and MPV remained consistently above the listed thresholds in all patients

### Hematological Findings

Macrocytic anemia, macrothrombocytopenia, neutropenia, and lymphopenia were universally present (Table 1). Patients received blood and platelet transfusions as needed. Vitamin B12, folic acid, and iron stores markers were within normal ranges (Table S1).

### Immunoglobulin Abnormalities

Quantitative immunoglobulin (Ig) analysis revealed several abnormalities (Table 2): Total IgG levels were elevated in three patients (P3, P5, P7) and within the reference range for the rest. IgG subclass analysis showed abnormal distribution patterns: IgG1 levels were elevated in P3 and P6; IgG2 was markedly decreased in P4 but significantly elevated in P5; and IgG3 levels were elevated in P1, P2, and P3. IgG4 levels were within reference ranges for all patients. IgA and IgM levels were consistently decreased in all patients, ranging from 7.5 to 28 mg/dL and from 10 to 36 mg/dL, respectively.

**Table 2:** Immunoglobulin levels, vaccine-specific antibodies, and lymphocyte subpopulations

Patient characteristics	P1	P2	P3	P4	P5	P6	P7	Reference range		
Age (years)	5	2.5	5	3	10	4	6			
Immunoglobulins								2-4y	4-7y	7-10y
IgG (mg/dL)	956	985	1750	800	1060	800	1300	295-1,156	386-1,470	462-1,682
IgG1 (mg/dL)	554	529	1000	510	605	420	N/A	158–721	209–902	253-1,019
IgG2 (mg/dL)	98	63.5	160	<2	417	125	N/A	37–184	44–316	54–435
IgG3 (mg/dL)	127	132	310	100	120	60	N/A	39–176	10.8–94.9	8.5–102.6.5.6
IgG4 (mg/dL)	51	28.5	32	45	4	2	N/A	17.0–84.7.0.7	≤ 81.9	1.0–108.7.0.7
IgA (mg/dL)	14.6	7.5	11	8	31	11	27	≤ 49.1	29–256	34–274
IgM (mg/dL)	17.3	12	18	11	26	10	15	27–246	37–224	38–251
IgE (IU/mL)	24.3	4	10	8	25	2	N/A	37-184		
Specific antibodies										
Tetanus toxoid (IU/ml)	0.011	0.01	0.33	0.262	0.173	N	0.063	> 0.5^A^, > 0.1^B^		
Pneumococcus (mg/dL)	3.5	0.6	4.4	2.6	16	N	13	1–27*		
Measles (AU/ml)	< 13.5	< 13.5	20	180	N/A	N	<13.5	**>16.5 AU/ml		
Lymphocyte subpopulations(% Positive cells in gated lymphocytes)								1–3 y	4–6 y	7–10
CD3 (cells x 10^3/µl)	0.90(88%)	1.26(83%)	1.03(78%)	1.38(82%)	1.38(86%)	1.1(60%)	1.2(85%)	1.02–4.7	0.99–3.9	0.81–3.13
CD4 (cells x 10^3/µl)	0.44(43.5%)	0.74(49%)	0.56(45%)	0.79(46%)	0.71(44%)	0.6(33%)	0.6(42%)	0.48–2.8	0.42–1.9	373.2–1920
CD8 (cells x 10^3/µl)	0.38(37%)	0.45(30%)	0.39(30%)	0.54(32%)	0.6(38%)	0.5(26%)	0.4(28%)	0.26–1.6	0.23–1.5	0.21–1.3
CD19 (cells x 10^3/µl)	0.06(6%)	0.1(6.5%)	0.12(9%)	0.07(4%)	0.05(3%)	0.08(4%)	0.08(5%)	0.23–1.7	0.16–1.2	0.11–1.6
CD56/CD16 (cells x 10^3/µl)	0.04(3.6%)	0.13(8%)	0.13(10%)	0.17(10%)	0.1(6%)	0.5(26%)	0.08(5%)	0.06–0.66	0.06–0.66	0.06–0.66

P, patient; N, non-immunized N/A, not available; ^A^ >0.5, short term protection ^B^ >1.0, long term protection

* <0.97 mg/dL poor response, 0.97–4.09 mg/dL modest response, 4.1–18.09.1.09 mg/dL intermediate, moderate 18.1–27 mg/dL moderate, > 27 mg/dL robust response

** <13.5 AU/ml negative, 13.5–16.5 borderline, > 16.5 positive

### Specific Antibody Responses

Functional antibody responses to vaccination were impaired in most patients (Table 2): Tetanus toxoid antibody levels were below protective levels in patients P1, P2, and P7 despite age-appropriate vaccination. Anti-pneumococcal antibody responses were classified as poor in patients P1, P2 and P4 (3.5, 0.6, and 2.6 mg/dL, respectively). Measles antibody titers were negative in patients P1, P2, and P7, indicating an impaired response to live attenuated viral vaccines.

### Lymphocyte Subpopulations

Lymphocyte immunophenotyping revealed a consistent pattern of B-cell lymphopenia (Table 2). Total B-cell (CD19+) counts were markedly reduced, ranging from 0.06 to 0.12 cells x 10^3/µl. The percentage of B-cells among gated lymphocytes was correspondingly low (4–9%). T-cell proportions were normal; absolute counts mildly reduced in the context of global lymphopenia. In contrast to the profound B-cell deficiency, all seven patients demonstrated normal T-cell receptor excision circles (TREC) levels at newborn screening. Natural killer (NK) cell counts were normal in most patients, apart from P1 (0.04 cells x 10^3/µl).

### B Cell Subpopulations and KREC

Detailed analysis of B-cell subsets revealed heterogeneous alterations in maturation (Table 3): The naive B-cell compartment was variably affected (15–41% of total B-cells). Transitional B-cells were relatively preserved (4–12% of the B-cell population). Switched memory B-cells showed an aberrant distribution, being reduced in P1 and P3 (1.5% and 6%) but increased in P2, P4, P6, and P7 (30–36%). IgM memory/marginal zone B-cells were decreased in most patients (0.4–4%). Two patients (P2 and P6) exhibited a marked expansion of plasmablasts(> 30% of total B-cells). While certain B-cell subsets appeared proportionally increased, these percentages represent a mathematical artifact of the profound global B-cell lymphopenia; the absolute counts of all subpopulations (i.e., switched memory B-cells [range: 2–18 cells/µL]) remained significantly below age-specific reference ranges. KREC levels (Table 3) were variable; although they remained above the diagnostic threshold for complete B-cell aplasia, the absolute counts (6–50 copies/µL) were significantly below the 5th percentile for age-matched healthy controls. [17, 18].

**Table 3:** B cell subpopulation

Age (years)	P1	P2	P3	P4	P5	P6	P7
	5	2.5	5	3	10	4	6
KREC (copies per microliter blood)	14	50	6	13	46	24	N/A
**B cells populations **(% of CD19)							
**Naïve B cells**	41%	27%	53%	44%	47%	26%	27%
**Transitional B cells**	41%	30%	15%	29%	20%	24%	39%
**IgM memory/marginal B cells**	7%	4%	10%	12%	4.5%	6%	7%
**Switched memory B cells**	5%	6.5%	7.5%	5%	17.5%	5%	13%
**IgD only memory B cells**	0.4%	0.6%	3%	2%	0.3%	1.5%	0.7%
CD27⁻IgM⁻ double negative B cells	3.5%	2.7%	4%	3.5%	3.5%	4%	2.7%
**Plasmablasts**	1.5%	30%	6%	4%	7%	33%	10%
**CD27+**	N/A	35	19%	30%	N/A	31%	36%

P, patient; N/A, not available; KREC, Kappa-deleting Recombination Excision Circles

## Discussion

This study highlights the pleiotropic effects of a GALE variant beyond galactosemia, revealing a profound impact on the immune system. Our cohort of seven pediatric patients, all homozygous for the p.R51W GALE variant, exhibited a consistent inborn error of immunity (IEI) characterized by B-cell lymphopenia, low IgA/IgM, abnormal B-cell subset distribution, and impaired vaccine responses. These findings represent the largest cohort to date documenting immunological abnormalities in GALE deficiency, significantly extending the limited prior data [16].

The finding of detectable KREC levels across our cohort, despite profound B-cell lymphopenia, argues against an absolute developmental block in the bone marrow. However, as absolute KREC counts were significantly below the age-specific 5th percentile [17, 18], our data indicate a profound quantitative reduction in central B-cell output. While a full spectrum of maturation stages remains present, indicating no absolute block in differentiation, the failure to maintain a stable, quantitatively adequate B-cell pool suggests a fundamental defect in cellular homeostasis.

**Fig. 1:**
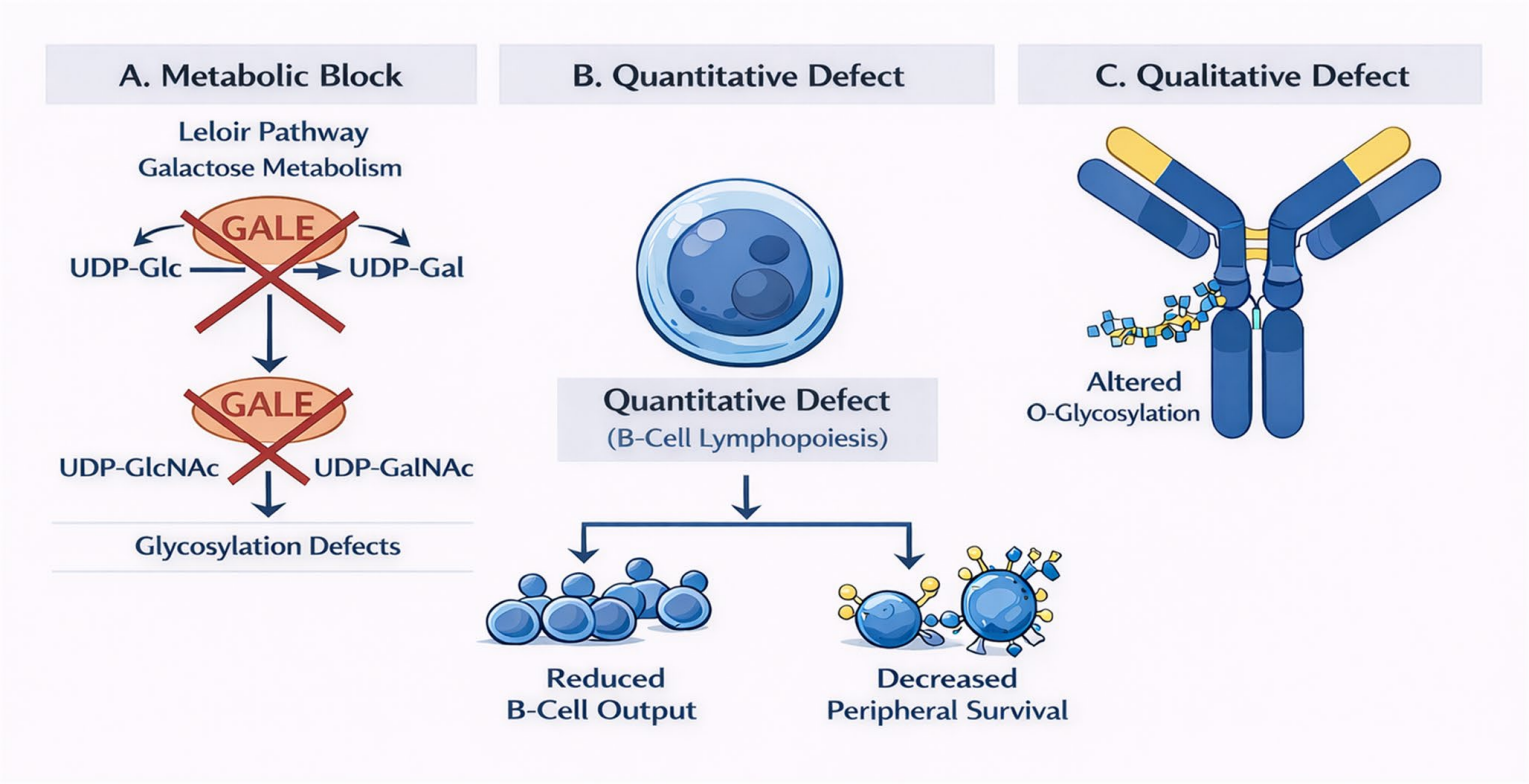
Proposed Pathophysiology of the GALE p.R51W Variant. (**A**) Metabolic and Glycosylation Crosstalk. The p.R51W variant impairs both galactose metabolism via the Leloir pathway and the interconversion of nucleotide sugars (UDP-Gal and UDP-GalNAc). This dual defect is predicted to disrupt the availability of critical donors required for downstream protein and lipid glycosylation (**B**) Quantitative B-cell Defect. Impaired glycosylation during lymphopoiesis may lead to reduced central B-cell output, consistent with absolute KREC counts below the age-specific 5th percentile. The resulting global B-cell lymphopenia likely reflects a combination of decreased bone marrow production and disrupted peripheral homeostasis (**C**) Qualitative Humoral Dysfunction. *GALE*-associated glycosylation defects may alter humoral immunity, resulting in impaired vaccine-specific antibody responses. In addition, elevated serum IgG3 may reflect compensatory plasmablast differentiation or altered IgG3 half-life secondary to aberrant hinge-region O-glycosylation

In addition to these quantitative deficits, the uniformly low IgA and IgM levels, the preserved or elevated IgG (specifically IgG3), and the impaired vaccine-specific antibody responses point to a concurrent qualitative defect in B-cell function. We propose that the p.R51W GALE variant represents a hematopoietic stress phenotype where reduced central output is exacerbated by impaired peripheral survival (Fig. 1). This cellular pattern mirrors abnormalities reported in other glycosylation disorders affecting the immune system, such as PGM3-CDG and JAGN1-CDG [19–22]. In these conditions, as in GALE deficiency, the primary immunological burden results from a failure to maintain a stable and functional B-cell compartment despite the presence of early progenitors.

The elevation of serum IgG3 in the context of GALE deficiency presents a biochemical paradox, as IgG3 stability relies on UDP-GalNAc-dependent O-glycosylation of the hinge region, a substrate potentially depleted in these patients. Two potential mechanisms may account for this observation. First, the skewed B-cell phenotype suggests a compensatory differentiation toward plasmablasts. In the context of profound B-cell lymphopenia, the surviving pool may be preferentially driven toward plasmablast generation to maintain basic humoral immunity. While high plasmablast percentages are partly a mathematical artifact of the low total B-cell count, they reflect a differentiation shift that favors rapid antibody production. Second, aberrant O-glycosylation of the IgG3 hinge may paradoxically prolong its serum half-life by interfering with proteolysis or receptor-mediated clearance. If the p.R51W variant results in a “leaky” or partial substrate deficiency, incomplete glycosylation of the unusually long IgG3 hinge may shield the protein from standard degradative pathways. Future studies utilizing mass spectrometry for global glycomic profiling will be essential to directly characterize these isotype-specific glycosylation patterns and confirm the underlying biochemical mechanism.

The GALE variant identified in our patients exhibits biochemical characteristics consistent with a congenital disorder of glycosylation (CDG). As GALE provides essential UDP-sugars for O-linked glycosylation [23], defective activity could disrupt B-cell receptor assembly, antibody secretion, and cell survival [14, 15]. However, the clinical course of our cohort was characterized predominantly by recurrent viral respiratory infections without invasive bacterial disease, sepsis, or a requirement for immunoglobulin replacement (IVIG). This “leaky” phenotype indicates that the small peripheral B-cell pool, potentially supported by the preserved IgG3 levels, provides sufficient humoral defense to prevent life-threatening complications in early childhood. The selective reduction in IgM and IgA production likely contributes to the recurrent viral infections observed in our cohort, while the relatively preserved IgG (particularly the IgG3 subclass) may provide partial protection against bacterial pathogens.

Reductions in T-cell counts were observed; however, these were proportional to the overall lymphopenia and lacked clinical features suggestive of T-cell immunodeficiency, such as opportunistic infections or severe viral disease. Importantly, normal TREC levels on newborn screening support intact thymic output and argue against a primary T-cell developmental defect. While broader hematologic abnormalities like macrothrombocytopenia were present, their full characterization lies beyond the scope of the present immunological analysis.

While the cellular mechanism of B-cell failure aligns with that of some other glycosylation disorders, the clinical presentation of our cohort differs significantly from established IEIs, such as MOGS-CDG (Mannosyl-oligosaccharide glucosidase deficiency) and MAGT1-CDG, also known as X-linked immunodeficiency with magnesium defect, Epstein-Barr virus (EBV) infection, and neoplasia (XMEN). MOGS-CDG presents with profound hypogammaglobulinemia [24], while XMEN is a combined immunodeficiency characterized by a specific susceptibility to chronic EBV infection and EBV-associated lymphoproliferation [25]. Unlike these global processing defects, our findings in GALE deficiency indicate a predominantly B-cell specific abnormalities and an absence of invasive infections, dysmorphism, autoimmunity, or severe atopy. This further distinguishes the p.R51W GALE variant as a more lineage-restricted hematopoietic phenotype.

Given the young age of our patients, we cannot exclude the possibility of future decline. Seo et al. [9] described three patients with this variant who underwent hematopoietic stem cell transplantation (HSCT) for thrombocytopenia and are now in their 20 s and 30 s with normalized lymphocyte counts. This contrasts with the single case report of an un-transplanted older patient who exhibited persistent B-cell lymphopenia [16]. While HSCT with GALE-competent donor cells may restore internal glycosylation pathways and normalize lymphocyte counts, its necessity for immunological indications remains speculative. Management should prioritize conservative supportive care, including close immunological monitoring and optimized vaccination. Reserved for life-threatening or refractory cases, HSCT remains a secondary consideration behind first-line interventions such as IVIG or prophylactic antibiotics where clinically indicated.

### Limitations

The primary limitations of this study include the small cohort size and the focus on a single, regionally prevalent *GALE* variant (p.R51W). The absence of a control group consisting of other *GALE* genotypes or Leloir pathway defects (e.g., GALT deficiency) makes it difficult to definitively isolate genotype-specific effects. Furthermore, while normal TREC levels are encouraging, the absence of high-resolution glycomic profiling and functional T-cell assays, such as proliferation and activation studies, means that subtle qualitative T-cell defects cannot be entirely ruled out. Future research utilizing mass spectrometry and functional cellular assays across a broader range of *GALE* variants will be essential to fully characterize the impact of this inborn error of immunity on lymphocyte homeostasis.

### Clinical Implications

Our findings expand the clinical spectrum of GALE-associated disorders, necessitating comprehensive immunological assessment for all patients regardless of their metabolic status. While our cohort focuses on the p.R51W variant, we recommend systematic evaluation of the immunological status across all GALE deficiency genotypes. Management should prioritize a conservative, supportive approach, including close monitoring and vaccination review, reserving IVIG, antibiotic prophylaxis, or HSCT for refractory or life-threatening cases. Longitudinal follow-up is essential to monitor for potential immunological deterioration and to guide the escalation of therapy as these patients transition into adulthood.

## Supplementary Information

Below is the link to the electronic supplementary material.

Supplementary File 1 (DOCX 23.3 KB)

## Data Availability

The datasets generated during and/or analyzed during the current study are available from the corresponding author on reasonable request.
