# Supplementary material for: Immunological Manifestations in GALE Deficiency: Extending the Spectrum Beyond Thrombocytopenia and Galactosemia
Source: J Clin Immunol. 2026 Apr 16;46(1):55. doi: 10.1007/s10875-026-02018-w (PMC13201354; doi:10.1007/s10875-026-02018-w)
Supplement: Supplementary file 1 — (DOCX 23.3 KB) [file 10875_2026_2018_MOESM1_ESM.docx]

**Supplementary Information**

**Immunoglobulin and haematology**

Reference intervals were derived from local laboratory standards.

**Flow cytometry:** Peripheral blood WBCs were stained with monoclonal antibodies against surface T- and B-lymphoid markers using standard procedure. Briefly, 100 ml of peripheral blood was incubated for 30 min with fluorescent monoclonal antibodies and exposed to BD FACS lysis buffer to remove red blood cells. Stained cells were washed twice with PBS supplemented with 0.2% bovine serum albumin and 0.02% sodium azide and analyzed using a FACSScan II flow cytometer equipped with BD FACSDiva Software, version 6.1.2 (BD Biosciences; San Jose, CA). A number of eight-color cell staining protocols were assembled using the following monoclonal antibodies: CD3-PerCP-Cy5.5, CD3-APC, CD4-PC7, CD5-PerCP-Cy5.5, CD7-Horizon V450, CD8-APC, CD8-PB, CD16-Horizon V500, CD19-PE-Cy7, CD19-BV510, CD21-PE, CD23-PerCP-Cy5.5, CD27-BV510, CD38-FITC, CD45-APC-H7, CD45RA-BV421, CD56-PE, CD57-FITC, CD197-PE, as well APC-labeled antibody against IgM and BV421-labeled antibody against IgD. The antibodies labeled with FITC, PE, and PE-Cy7 fluorochromes (except for anti-CD21-PE, anti-CD197(CCR7)-PE reagents), APC-labeled anti-CD3 and anti-CD8 antibodies were purchased from Immunotech SAS (Beckman Coulter; Marceille, France). All the other monoclonal antibody reagents were from BD Biosciences (San Jose, CA) or a BD PharMingen subsidiary (San Diego, CA).

The subsets of CD3+, CD4+, CD8+, and CD57+ T cells, CD3-/CD16+ and/or CD56+ NK cells, and CD19+ B cells were routinely detected and counted. In addition, advanced analysis of peripheral blood B lymphocytes was performed. The subpopulations Analysis of gated CD19+ B lymphocytes included detection and differential count of CD23-/CD27-/CD38+/IgD+/IgM+ transitional B cells, CD23+/CD27-/CD38-/IgD+/IgM+ naïve B cells, CD23-/CD27+/CD38-/IgD+/IgM+ non-switched memory/marginal zone B cells, CD23-/CD27+/CD38-/IgD-/IgM- switched memory B cells, IgD-only positive (IgD+/IgM-) B cells, IgM-/CD27- double-negative (DN) B cells, and plasmablasts (B–lineage cells with a strong expression of CD38) were counted.

**Newborn screening program in Israel**

The Newborn Screening (NBS) Program in Israel is a nationwide initiative in which all samples are transferred and analyzed at a single laboratory. On average, results are reported by the fourth day of life.

The National NBS Program works in close collaboration with all metabolic clinics across the country. These clinics receive referrals of infants with positive screening results. For any positive NBS result indicating a potential disorder, a rapid confirmatory molecular test is available using a fresh EDTA blood sample.

**Galactosemia newborn screening**

In Israel, newborn screening for galactosemia includes simultaneous measurement of galactose-1-phosphate (Gal-1P) alongside other analytes routinely detected by flow injection analysis–tandem mass spectrometry (FIA-MS/MS). This serves as the **first-tier test,** followed by a **second-tier GALT enzyme assay** for samples showing elevated Gal-1P levels [1].

The Israeli NBS algorithm can also identify UDP-galactose-4′-epimerase (GALE) deficiency in individuals from consanguineous Arab Bedouin kindred. These cases are characterized by elevated Gal-1P levels, normal GALT enzyme activity, and the *GALE* c.151C>T (p.R51W) variant.

**Kappa-deleting Recombination Excision Circles**

The Israeli NBS program for severe combined immunodeficiency (SCID) utilizes the commercial EnLite™ Neonatal TREC kit (PerkinElmer, Turku, Finland) according to the manufacturer’s instructions. Kappa-deleting recombination excision circle (KREC) measurement was conducted and utilized the analytical performance of the **EONIS™ Q** real-time PCR platform and associated assay(Revvity; <https://www.revvity.com/product/eonis-q96-2044-0020>). Results were reported as [copies per microliter blood].

**References**

1. Daas S, Abu Salah N, Anikster Y, Barel O, Damseh NS, Dumin E, et al. Addition of galactose-1-phosphate measurement enhances newborn screening for classical galactosemia. *J Inherit Metab Dis*. 2023;46(2):232–242. doi:10.1002/jimd.12580

Table S1- Elaborate haematological laboratory results

These parameters were collected to exclude nutritional deficiencies or autoantibodies as contributors to cytopenias

|  | P1 | P2 | P3 | P4 | P5 | P6 | P7 |
| --- | --- | --- | --- | --- | --- | --- | --- |
| Folic acid:  ng/ml (3.1-20) | 11.9 | 5.3 | 5.7 | 22.3 | 17 | 4 | 6.6 |
| Vitamin B12: pg/ml(135-911) | 430 | 1604 | 575 | 437 | 604 | 724 | 397 |
| Ferritin: ng/ml | 155.2 | 77.2 | 164 | 152.5 | 128.6 | 6.6 | 570 |
| ABO/Rh | B+ | O+ | O+ | B+ | O+ | B+ | B+ |
| Direct Coombs  (antibody screen) | negative | negative | negative | negative | negative | negative | negative |
